# Swimming Speed of Larval Snail Does Not Correlate with Size and Ciliary Beat Frequency

**DOI:** 10.1371/journal.pone.0082764

**Published:** 2013-12-18

**Authors:** Kit Yu Karen Chan, Houshuo Jiang, Dianna K. Padilla

**Affiliations:** 1 Biology Department, Woods Hole Oceanographic Institution, Woods Hole, Massachusetts, United States of America; 2 Department of Applied Ocean Physics and Engineering, Woods Hole Oceanographic Institution, Woods Hole, Massachusetts, United States of America; 3 Department of Ecology and Evolution, Stony Brook University, Stony Brook, New York, United States of America; University of Hull, United Kingdom

## Abstract

Many marine invertebrates have planktonic larvae with cilia used for both propulsion and capturing of food particles. Hence, changes in ciliary activity have implications for larval nutrition and ability to navigate the water column, which in turn affect survival and dispersal. Using high-speed high-resolution microvideography, we examined the relationship between swimming speed, velar arrangements, and ciliary beat frequency of freely swimming veliger larvae of the gastropod *Crepidula fornicata* over the course of larval development. Average swimming speed was greatest 6 days post hatching, suggesting a reduction in swimming speed towards settlement. At a given age, veliger larvae have highly variable speeds (0.8–4 body lengths s^−1^) that are independent of shell size. Contrary to the hypothesis that an increase in ciliary beat frequency increases work done, and therefore speed, there was no significant correlation between swimming speed and ciliary beat frequency. Instead, there are significant correlations between swimming speed and visible area of the velar lobe, and distance between centroids of velum and larval shell. These observations suggest an alternative hypothesis that, instead of modifying ciliary beat frequency, larval *C. fornicata* modify swimming through adjustment of velum extension or orientation. The ability to adjust velum position could influence particle capture efficiency and fluid disturbance and help promote survival in the plankton.

## Introduction

Many small planktonic organisms use cilia to propel themselves or to generate feeding currents, including larval stages of numerous marine invertebrates [1], [2], [3], [4]. Larval cilia are often arranged in bands or in loops around long slender extensions [5], [6]. Because of small size and relatively low swimming speeds, planktonic larvae, in general, operate in low Reynolds number environments in which viscous force dominates [7]. Hence, the thrust exerted by a larval body on the surrounding water is proportional to the length of the cilia, the frequency and synchrony of ciliary beat, and the total length of the ciliated bands relative to body size [2], [8], [9]. Small scale changes in ciliary motion could potentially impact population-scale dynamics by affecting swimming [10], [11] and food capture [2], [12], and thus, survival and dispersal of larvae [4], [13].

Veliger larvae of gastropods have long compound cilia on their velar lobes, long extensions used for both swimming and feeding [2]. These cilia are arranged in prototroch and metatroch bands surrounding a food groove. Particles are captured through filtration or direct interception [12]. Because of the dual function of the ciliated velum, there are likely tradeoffs between particle capture and swimming [3], [14]. Although an increase in swimming speed could increase area searched for food, faster swimming may not lead to an increase in particle capture. On the contrary, tethering, e.g., by weight of larval shell or a mucus strand, may impede motion, and could enhance filtering efficiency [15], [16].

Histological and immunocytochemical studies have demonstrated various neural innervations of the velar lobes and velar musculature of larval gastropods [17], [18]. Exposure to various neurotransmitters have been shown to affect depolarization across the ciliated membrane, causing changes in ciliary beat frequency or inducing ciliary arrest [19], [20]. These changes in ciliary motion are associated with changes in larval vertical distribution, e.g., addition of serotonin caused larval mud snails, *Ilyanassa obsolete*, to concentrate toward the top of a water column [21]. These studies highlight that veliger larvae can control ciliary motion and the musculature associated with the velum at a fine scale. However, to date, few studies have simultaneously observed these chemically induced ciliary motion adjustments and the organismal level response expressed as changes in swimming behaviors.

The Atlantic slipper snail, *Crepidula fornicata*, provides a model system for developmental and larval biology [22]. This protandrous hermaphroditic gastropod lays egg capsules from which planktotropic veliger larvae hatch. Developmental ecology and settlement behaviors of *C. fornicata* veligers are well described [23], [24], [25], [26]. Serotonin-, catecholamine- and FMRFamide-containing cells innervate the velum of *C. fornicata* and have been suggested to be involved in velum withdrawal, swimming, and feeding [18]. Laval *C. fornicata* swimming speed differs between families but does not correlate with growth rate measured by shell length [27]. This lack of relationship between swimming and growth, and by extension food consumption, supports the idea that there is functional tradeoff such that larvae are “good eaters but poor swimmers” [3]. However, it is unclear how veliger larvae modify their swimming speeds such that larval swimming speed can vary from 0.8 to 2.4 mm s^−1^one day post hatching [27]. Small-scale, high-resolution, high-speed videography has been applied to study behaviors of other zooplankton, e.g., jumping and resulted fluid motion in copepods [28], [29]. By applying such videography techniques to larval *C. fornicata* with well-described neuromuscular control, we aim to quantify the relationship between ciliary activity, velum arrangement, and whole-organism motion.

## Materials and Methods

### Larval rearing

Sexually mature adult *Crepidula fornicata* were collected from Crab Meadow Beach, Northport, NY (40.929731°N, −73.327256°W) in January 2013. No permissions or permits were required for collection of these animals at our collection site as it is a public access beach and this species is not regulated in New York waters. This species is one of the most abundant gastropods on Long Island shores and is not endangered or protected here or elsewhere within its native range. Male-female pairs were kept in 500 ml containers in the laboratory at Stony Brook University and were warmed from 10°C to 18°C over a two week period, and fed concentrated phytoplankton (Shellfish Diet, Reed Mariculture) daily. Egg masses were collected from multiple females and allowed to hatch in 0.2 µm filtered seawater. The larvae used in this experiment were collected from multiple parents on March 14, 2013 and sent to the Woods Hole Oceanographic Institution. Larvae were kept in glass containers filled with 0.22 µm filtered seawater at a density of 0.2 individual ml^−1^ at 18°C in 12∶12 light dark cycle. Larvae were fed 20,000 cells ml^−1^ *Isochrysis galbana* (CCMP strain 1324) every day. Algal concentration was determined by heamocytometer counts. Larvae were individually pipetted into clean containers with filtered seawater every 3 days.

### Video observations

**Figure 1:**
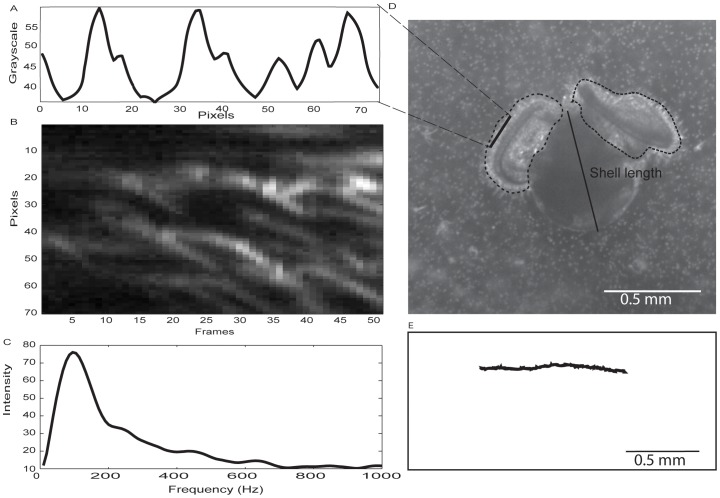
Video analysis approach taken to quantify swimming speed and ciliary beat frequency simultaneously. a) intensity (grayscale value between 0 and 255) for each pixel along a small region of interest highlighted on a 14 day old larval *Crepidula fornicata* as shown in d). b) Time series (50 frames at 2000 fps) of the intensity of each pixel along the region of interest. Bright areas indicate the passage of a cilium. c) Results from Fast Fourier Transform (FFT, Matlab) showing the peak frequency to be the ciliary beat frequency. d) Dotted line outline the velar area which affected swimming speed. e) Swimming trajectory of larva shown in d) over 0.95 s at 500 fps extracted using MTrack2.

Swimming and water flow visualization were made in small tissue culture flasks (Corning, surface area of 25 cm^2^) with approximately 70–80 larvae and sufficient 3 µm diameter polystyrene trace particles to make the water slightly cloudy. The setup was illuminated with collimated light supplied by a 4×6 array of super bright white LEDs. High-speed (2000 Hz) high-resolution (1024×1024 pixels) video recordings were made with a PhotronFastcam SA3 120K monochrome camera, fitted horizontally with a continuously adjustable bellows and a reversed 35 mm Nikkon camera lens. Such a setup generates a vertically oriented field of view. The two fields of view used were approximately 1.4×1.4 mm^2^ and 2.7×2.7 mm^2^ respectively (Fig. 1). This high-speed high-magnification imaging is necessary for resolving both the swimming motion of the larval body, position of velar lobes, and beating of cilia on the velum. Observations were repeated five times throughout development (2–19 days post hatching).

### Quantifying organismal and ciliary motion

Video clips with individuals clearly intersecting the focal plane were selected for further analysis (Table S1). To extract swimming speed, we imported the video clip as an image sequence at a lower frame rate (500 Hz) to the open source software Fiji to reduce frame rate noise [30]. We first removed background noises by applying a Gaussian blur to each frame and thresholded for brightness (grayscale) of the individual larva. We then used the tracking routine MTrack2 to extract the total length, displacement, and duration of the trajectory of each individual [31]. We computed the speed of the larva as the total length of the path divided by the total duration of observation (Fig. 1). We also measured shell lengths and areas of velar lobes from the frame that was in the middle of the whole image sequence. To explore the relationship between swimming speed and the orientation of vela, we also located the centroid of each velum and larval shells and computed the distance between them.

To investigate the relationship between average swimming speed and ciliary activity, we quantified the ciliary beat frequencies of all the individuals observed 14 days post fertilization (n = 20). To avoid selection bias, we adapted the approach of Sisson et al. [32] and Dimova et al. [33] to automate the analysis of ciliary beat frequency. Because light intensity changes as a cilium travels through a pixel over time, we recorded the intensity of a small region of interest on the ciliated band for 100 frames at the middle of the observation duration. We then performed a Fast Fourier Transform on the modified intensity, i.e., the intensity value of each pixel minus the global mean to enhance contrast, to extract the peak frequency (Fig. 1). To validate the effectiveness of this automated approach, we compared the outcome of this analysis with conventional photometry method where ciliary beat frequency is estimated by following the displacement of the tip of a single protochoral cilium over 100 frames at 2000 Hz. We performed the visual analysis on three individuals that were chosen based on their swimming speeds to include a representative range, close to the population average, maximum, and minimum values. To test the repeatability of the automated approach, we also performed the analysis on three different regions of interests and at different five time intervals for the same individual.

### Statistical analysis

We tested for variations in size-normalized swimming speed with age with an ANOVA. Data were square root transformed to meet the assumption of normality for ANOVA. Larval orientation varied between observations, thus, part of the velar lobes were obstructed at times. Therefore, we used the velar lobe with larger visible area for further statistical analysis, i.e., the maximum velar size or separation distance between velum and shell is only for one side (left or right) of a larva. To investigate the relationship between swimming speed, larval size, area of velum, separation distance between velum and shell, and ciliary beat frequency, we used Pearson's correlations. All the statistical analyses were conducted with the PASW 13.0.

## Results

### Novel applications of analytical techniques for larval motion

By applying high-speed, high-resolution microvideography, we simultaneously recorded organismal behaviors, velar position, and ciliary activity of freely swimming veligers of *Crepidula fornicata*. This approach made an explicit test of the relationship between ciliary beat frequency and swimming speed possible. We also demonstrated a novel application of ciliary beat frequency analysis previously used in medical studies [32]. This automated technique provided similar results when compared to traditional photometry analysis (paired sample t test, t_2,0.05_ = −2.828, *p* = 0.103). When three different regions of interest and five, non-overlapping time intervals of the same individual were analyzed, the resulting peak ciliary beat frequencies were the same.

### High variability in swimming speeds

**Figure 2:**
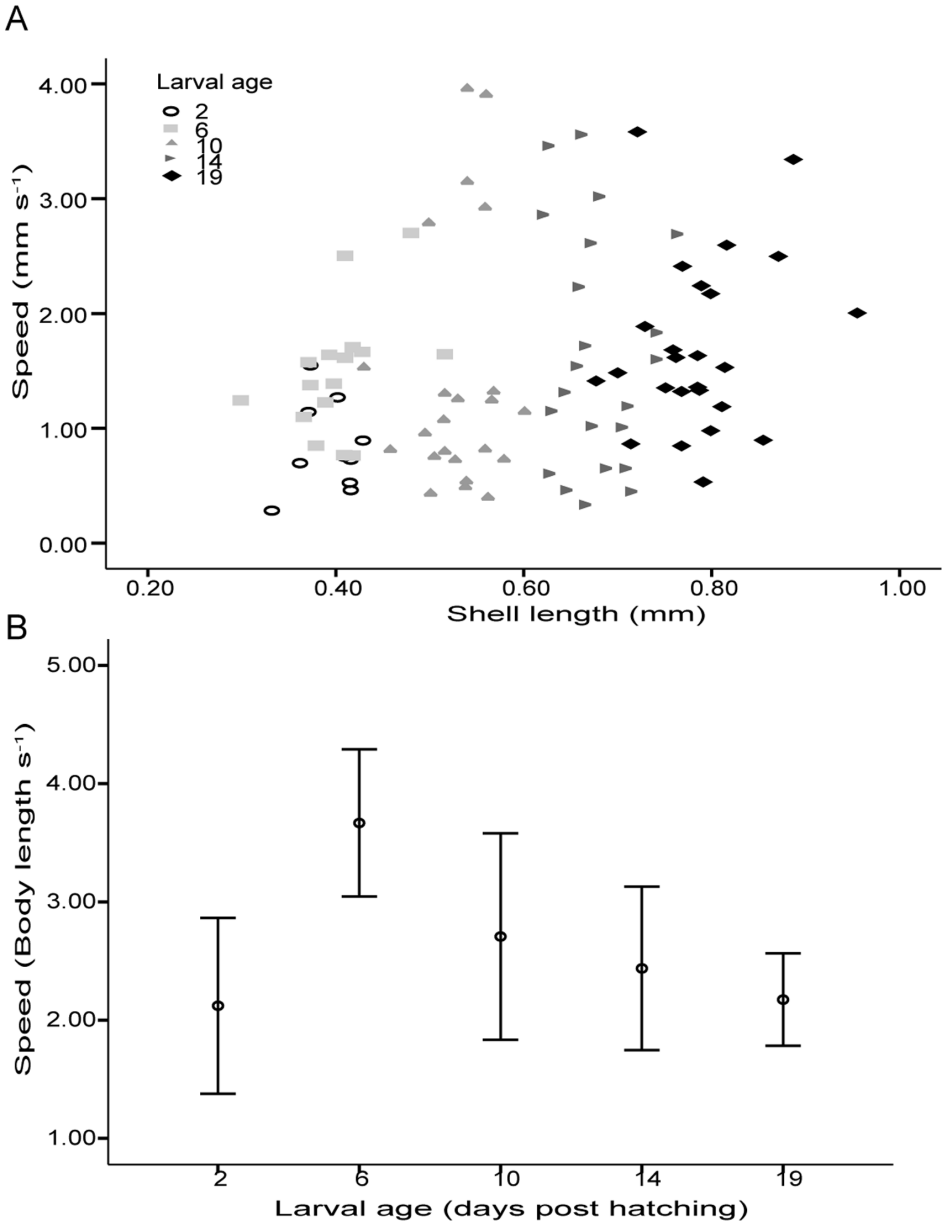
Average swimming speed of larval *Crepidula fornicata* from 2–19 days post hatching. a) Swimming speed varied over time but did not vary with shell length for any specific day of age. b) Normalized swimming speed varied significantly with age.

Veliger shell length (size) was positively correlated with larval age post hatching (Fig.2a, Pearson's Correlation, n = 96, *r* = 0.94, *p*<0.0001). When controlled for age, there was no significant correlation between swimming speed and shell length, i.e., for any age, larger larvae did not swim faster (Fig. 2, Partial Correlation, *r* = 0.036, *p* = 0.727). To test if there was an ontogenetic change in swimming speed, we expressed swimming speed as a function of body length, and compared the square root transformed speed observed on different dates with an ANOVA. The average swimming speed differed significantly with age (ANOVA, F*_1, 95_* = 3.233, *p* = 0.016). A plot of the average speed with 95% confidence intervals against larval age appeared hyperbolic (Fig. 2b), and a post-hoc Tukey's test confirmed that the average speed measured 6 days post hatching was significantly faster than those measured 2 and 19 days post hatching.

### Swimming speed not correlated with ciliary beat but with velum arrangement

**Figure 3:**
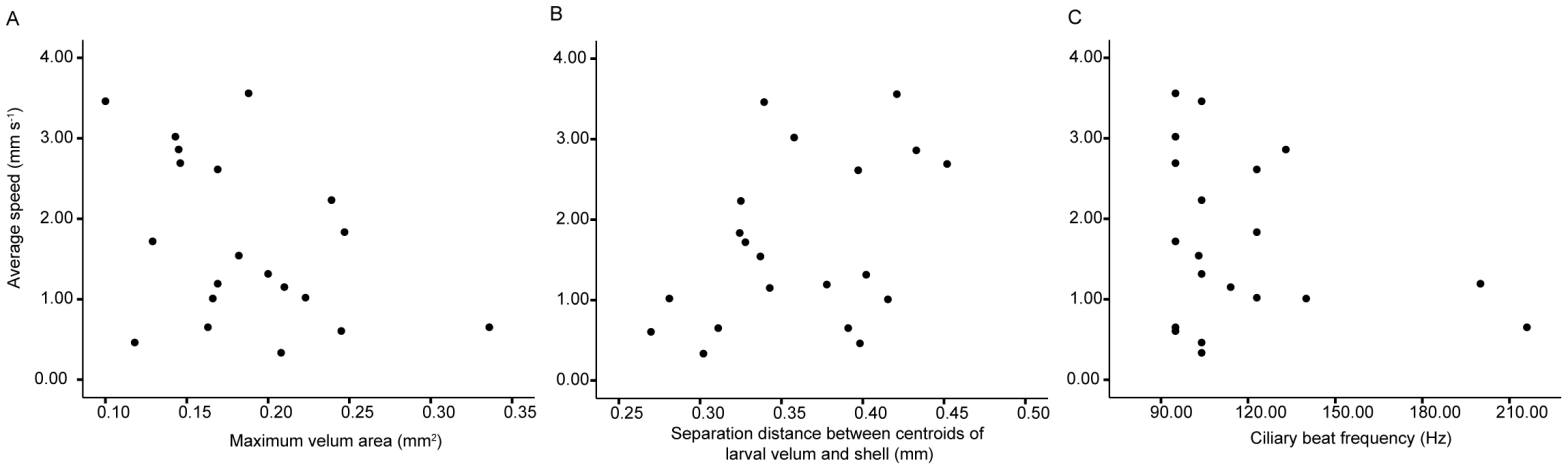
Average swimming speed correlated with velar arrangement but not ciliary beat frequency. a)Swimming speed significantly correlated with maximum area of velar lobes and b) separation distance between centroids of velum and larval shell. However, c) ciliary beat frequency did not have a significant relationship with average swimming speed for all 20 individuals observed 14 days post hatching.

Given that the automated ciliary beat frequency estimation techniques yielded similar results to those of the traditional photometry methods, we applied the automated techniques to all 20 events observed for 14 day old individuals. The ciliary beat frequency ranged between 95 and 216 Hz (Fig. 3c). There was no significant correlation between ciliary beat frequency and swimming speed (Pearson's correlation, *r* = −0.261, *p* = 0.126) or between ciliary beat frequency and shell length (Pearson's correlation, *r* = 0.334, *p* = 0.075).

**Figure 4:**
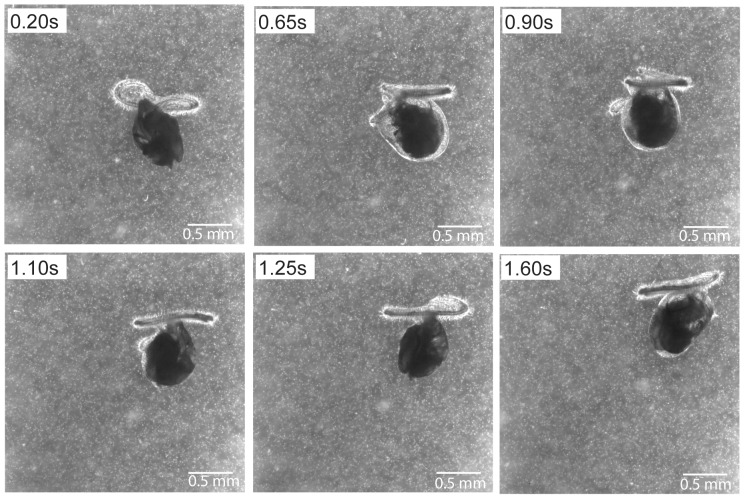
Larval *Crepidula fornicata* have a high degree of control of velar lobe extension and orientation. This 19 day old individual swam upward with only one of two velar lobes fully extended.

We also noted that extension and orientation of the velum differed between individuals and could change over the course of the observation. As an example of the variability in velum position, a 19 day old individual only fully extended one of the two velar lobes while swimming upwards, and flexed and bent the velum over a time scale of less than 0.5 s (Fig. 4, Video S1). In the 20 events observed for 14 day old individuals, the area of velum extended ranged between 0.1 to 0.34 mm^2^. There was a significant negative relationship between swimming speed and maximum velum area, i.e., velum extension (Pearson's correlation, *r* = −0.406, *p* = 0.038). Swimming speed increased with an increase in separation distance between the centroids of larval velum and shell. Faster swimming individuals had the velum spread further away from the shell (Pearson's correlation, *r* = 0.405, *p* = 0.038).

## Discussion

Ciliary activity affects larval abilities to capture food and swim, and in turn has implications for survival and dispersal. By modifying and applying high-speed high-resolution microvideography, we tracked the movement of freely swimming veliger larvae of *Crepidula fornicata*. We also demonstrated the use of automated image analysis techniques to simultaneously quantify ciliary motion. Despite the high variability in swimming speeds, there was no significant correlation between larval size or ciliary beat frequency and speed at a given age. However, we observed high levels of variability in velum extension and orientation, and these variations significantly correlated with swimming speeds. Such correlation between velum position and swimming speed is consistent with predictions of computational fluid dynamics (CFD) simulation that for a given amount of total propulsive force, a more spread-out force distribution dissipates less mechanical energy but results in a larger volume flux for capturing [34]. Hence, muscular control could have significant implications for larval movement.

Average swimming speed of larval *C. fornicata* peaked 6 days post hatching (Fig. 2b). Ontogenetic change in swimming behaviors has been observed in other planktonic larvae [35], [36]. One example is geotaxis, the response to gravity, which has been suggested to contribute towards larval settlement. Various veliger larvae were suggested to be more positively geotaxic as they age. However, Bayne [37] suggested that the reduction in swimming activity with age, rather than response to gravity, could also contribute towards the accumulation of larvae on the substratum [8]. The observed reduction in swimming speed for *C. fornicata* with age could be associated with competence for metamorphosis and settlement.

The high variability in swimming speed observed (from 0.28 to 3.95 body length s^−1^, Fig 2a) is consistent with the observations of Hilbish et al. [27]. They reported that there is a genetic basis to variability in swimming speed and growth rate, approximated by shell length, but the two factors do not covary. One of the hypotheses these authors proposed to reconcile this decoupling is that the faster swimming individuals have higher metabolic costs. Therefore, despite a larger area searched for food, the increased energy gain from food consumption is offset by the increased metabolic demand for locomotion and processing of ingested food. Alternatively, Hilbish et al. [27] suggest that the swimming and feeding are independent at the ciliary level due to the highly innervated-nature of the larval velum such that excitation does not spread to a large population of ciliated cells.

Observed swimming speed of *C. fornicata* correlated with velum extension and position, and therefore, supports the second hypothesis. Purcell [38] argues that the metabolic requirement of ciliary motion is low. Oxygen consumption, a proxy for metabolic cost, in ciliary-propelled larval bryozoans remains similar regardless of whether individuals are swimming or not [39]. Hence, increase in metabolic cost due to swimming alone is a less likely explanation for the lack of a significant relationship between swimming and growth. However, we do acknowledge that in some observations in *Paramecium*, energy demand for ciliary motion could be up to 50% [40]. Changing velum extension and orientation through muscular control could affect larval swimming speed without complementary changes in ciliary beat. Such independency may help veliger larvae balance the tradeoff between increasing filtering and swimming efficiency.

Swimming speed of *C. fornicata* 14 days post hatching did not correlate with ciliary beat frequency (Fig. 3c). There are several plausible, non-mutually exclusive explanations for this lack of correlation. First is that with the current videography and analytical techniques we may have missed other relevant ciliary activity. The ciliary beat frequencies we observed are limited to the longer, more visible cilia on the prototrochal band, and all observations were made on the same day. It is possible that the changes in ciliary motion took place in the opposing metatroch: it has been hypothesized that reduced ciliary beat frequency or increased frequency of ciliary arrest in the opposing ciliary bands could reduce swimming speeds [3]. Localized ciliary motion has also been reported in other gastropod veligers [19]. Given the small region of interest in our estimation, we could have missed short-term, localized changes in ciliary activity.

Aside from experimental artifacts, a second alternative explanation is that swimming by ciliary motion is not energy efficient with a maximum theoretical efficiency of <1% [9], [41]. Given larvae also operate at low Reynolds number, it is possible that changes in ciliary beat frequency for an individual of a certain size does not significantly affect swimming speed, due to low efficiency. Third, veliger larvae have rich neural innervations of the velar lobes and velar musculature [18], and hence there are alternative ways to influence swimming speed that are not related to ciliary beat. The observed correlation between velar lobe areas and separation distances between velum and shell suggest that changing velum extension and orientation is one such method.

Rapid changes in velum position also have implications for the intensity of fluid disturbance created by swimming larval *C. fornicata*, which could be variable over time. However, it is unclear whether the ability to change the velum is limited to this particular species or whether it is related to their increasingly asymmetric shell throughout development. Regardless, the observed variability in fluid disturbance could in turn affect the predator risk and larval survival [41], [42]. Future studies should investigate the potential tradeoff between maximizing the search area against area of influence by flow generated by individual larvae.

High-speed high-resolution microvideography enables detailed observation of freely behaving individuals. By applying this technique to veligers of *C. fornicata*, we demonstrated that larvae have highly variable swimming speeds that changed with age but were decoupled from shell size or ciliary beat frequency. Such individual level observations should be integrated with anatomical and biochemical studies to understand the underlying mechanisms for locomotion of smaller organisms, and with numerical modeling to explore ecological consequences of variations in individual locomotion.

## Supporting Information

Table S1 **Total length of trajectory, net displacement, duration of observation, average swimming speed and shell length of each individual observed over development from 2–19 days post hatching.**
(DOCX)

Video S1 **Larval** ***Crepidula fornicata*** **have a high degree of control of velar lobe extension and orientation.** This 19 day old individual swam upward with only one of two velar lobes fully extended. Video captured at 2000 fps and replayed at 200 fps.
(WMV)
