# Supplementary material for: Swimming Speed of Larval Snail Does Not Correlate with Size and Ciliary Beat Frequency
Source: PLoS One. 2013 Dec 18;8(12):e82764. doi: 10.1371/journal.pone.0082764 (PMC3867405; doi:10.1371/journal.pone.0082764)
Supplement: Table S1 — Total length of trajectory, net displacement, duration of observation, average swimming speed and shell length of each individual observed over development from 2–19 days post hatching. (DOCX) [file pone.0082764.s001.docx]

Table S1. Total length of trajectory, net displacement, duration of observation, average swimming speed and shell length of each individual observed over development from 2 - 19 days post hatching.

| **Larval age**  **(days)** | **ID.** | **Trajectory**  **length (mm)** | **Net displacement (mm)** | **Duration (s)** | **Speed (mms^-1^)** | **Shell length**  **(mm)** | **Average speed (± SE, mms^-1^)** |
| --- | --- | --- | --- | --- | --- | --- | --- |
| 19 | 1 | 0.03 | 0.03 | 0.02 | 1.68 | 0.76 | 1.71 ± 0.15 |
|  | 2 | 0.37 | 0.33 | 0.43 | 0.86 | 0.71 |  |
|  | 3 | 0.06 | 0.05 | 0.04 | 1.48 | 0.70 |  |
|  | 4 | 1.27 | 1.01 | 0.90 | 1.41 | 0.68 |  |
|  | 5 | 2.83 | 1.06 | 1.41 | 2.00 | 0.96 |  |
|  | 6 | 0.01 | 0.01 | 0.01 | 1.36 | 0.79 |  |
|  | 7 | 2.64 | 2.51 | 1.62 | 1.63 | 0.79 |  |
|  | 8 | 1.12 | 1.03 | 0.85 | 1.33 | 0.79 |  |
|  | 9 | 0.44 | 0.43 | 0.33 | 1.32 | 0.77 |  |
|  | 10 | 3.50 | 2.23 | 1.35 | 2.60 | 0.82 |  |
|  | 11 | 1.14 | 1.14 | 0.61 | 1.89 | 0.73 |  |
|  | 12 | 1.65 | 1.34 | 1.95 | 0.85 | 0.77 |  |
|  | 13 | 1.82 | 1.80 | 0.76 | 2.41 | 0.77 |  |
|  | 14 | 3.49 | 1.85 | 0.98 | 3.58 | 0.72 |  |
|  | 15 | 1.84 | 1.71 | 0.85 | 2.17 | 0.80 |  |
|  | 16 | 1.44 | 1.35 | 1.47 | 0.98 | 0.80 |  |
|  | 17 | 2.36 | 1.81 | 0.95 | 2.50 | 0.87 |  |
|  | 18 | 2.50 | 1.52 | 1.64 | 1.53 | 0.81 |  |
|  | 19 | 3.36 | 2.15 | 2.08 | 1.62 | 0.76 |  |
|  | 20 | 1.09 | 1.07 | 0.92 | 1.19 | 0.81 |  |
|  | 21 | 2.25 | 1.51 | 2.52 | 0.90 | 0.86 |  |
|  | 22 | 1.87 | 1.86 | 0.84 | 2.24 | 0.79 |  |
|  | 23 | 0.70 | 0.50 | 1.31 | 0.53 | 0.79 |  |
|  | 24 | 3.53 | 0.74 | 1.06 | 3.34 | 0.89 |  |
|  | 25 | 2.34 | 0.52 | 1.73 | 1.35 | 0.75 |  |
| 14 | 1 | 3.06 | 1.68 | 1.07 | 2.86 | 0.62 | 1.64 ± 0.22 |
|  | 2 | 0.22 | 0.19 | 0.14 | 1.54 | 0.66 |  |
|  | 4 | 2.95 | 0.85 | 1.84 | 1.60 | 0.74 |  |
|  | 5 | 1.83 | 1.00 | 0.52 | 3.56 | 0.66 |  |
|  | 6 | 1.28 | 0.26 | 1.27 | 1.01 | 0.70 |  |
|  | 7 | 2.04 | 1.14 | 1.11 | 1.83 | 0.74 |  |
|  | 8 | 0.33 | 0.17 | 0.13 | 2.61 | 0.67 |  |
|  | 9 | 1.72 | 1.62 | 0.77 | 2.23 | 0.66 |  |
|  | 10 | 0.91 | 0.46 | 0.89 | 1.02 | 0.67 |  |
|  | 11 | 3.66 | 0.37 | 1.36 | 2.69 | 0.76 |  |
|  | 12 | 1.46 | 0.18 | 1.11 | 1.32 | 0.64 |  |
|  | 13 | 2.87 | 0.42 | 1.67 | 1.72 | 0.66 |  |
|  | 14 | 1.27 | 0.61 | 2.76 | 0.46 | 0.64 |  |
|  | 15 | 0.79 | 0.63 | 0.69 | 1.15 | 0.63 |  |
|  | 17 | 0.49 | 0.39 | 0.76 | 0.65 | 0.69 |  |
|  | 18 | 2.15 | 0.29 | 0.62 | 3.46 | 0.63 |  |
|  | 19 | 2.85 | 1.25 | 0.95 | 3.02 | 0.68 |  |
|  | 20 | 0.40 | 0.35 | 0.88 | 0.45 | 0.71 |  |
|  | 21 | 1.65 | 1.21 | 2.73 | 0.61 | 0.63 |  |
|  | 22 | 0.34 | 0.14 | 1.01 | 0.33 | 0.66 |  |
| 10 | 1 | 1.64 | 1.50 | 0.42 | 3.96 | 0.54 | 1.44 ± 0.22 |
|  | 2 | 6.58 | 1.57 | 2.09 | 3.15 | 0.54 |  |
|  | 3 | 1.87 | 0.05 | 0.48 | 3.91 | 0.56 |  |
|  | 4 | 0.84 | 0.43 | 0.55 | 1.53 | 0.43 |  |
|  | 5 | 5.85 | 2.43 | 7.19 | 0.81 | 0.46 |  |
|  | 6 | 0.54 | 0.32 | 1.01 | 0.54 | 0.54 |  |
|  | 7 | 1.01 | 0.86 | 1.34 | 0.75 | 0.51 |  |
|  | 8 | 4.33 | 1.82 | 1.48 | 2.93 | 0.56 |  |
|  | 9 | 0.75 | 0.39 | 0.60 | 1.26 | 0.53 |  |
|  | 10 | 6.71 | 2.45 | 2.41 | 2.79 | 0.50 |  |
|  | 11 | 0.18 | 0.16 | 0.36 | 0.49 | 0.54 |  |
|  | 12 | 2.61 | 0.66 | 2.73 | 0.96 | 0.50 |  |
|  | 13 | 1.74 | 1.47 | 2.13 | 0.82 | 0.56 |  |
|  | 14 | 1.07 | 0.37 | 1.47 | 0.73 | 0.53 |  |
|  | 15 | 1.54 | 0.74 | 2.11 | 0.73 | 0.58 |  |
|  | 16 | 2.18 | 1.60 | 2.73 | 0.80 | 0.52 |  |
|  | 17 | 2.59 | 1.80 | 1.99 | 1.30 | 0.52 |  |
|  | 18 | 2.93 | 1.58 | 2.73 | 1.07 | 0.52 |  |
|  | 19 | 1.63 | 0.41 | 1.23 | 1.32 | 0.57 |  |
|  | 20 | 2.44 | 1.09 | 2.13 | 1.15 | 0.60 |  |
|  | 21 | 3.39 | 1.86 | 2.73 | 1.25 | 0.57 |  |
|  | 22 | 1.05 | 0.73 | 2.63 | 0.40 | 0.56 |  |
|  | 23 | 0.92 | 0.49 | 2.12 | 0.43 | 0.50 |  |
| 6 | 1 | 0.74 | 0.73 | 0.45 | 1.65 | 0.52 | 1.48±0.14 |
|  | 2 | 0.37 | 0.36 | 0.23 | 1.62 | 0.41 |  |
|  | 3 | 0.33 | 0.30 | 0.27 | 1.24 | 0.30 |  |
|  | 4 | 0.48 | 0.38 | 0.19 | 2.50 | 0.41 |  |
|  | 5 | 1.07 | 0.86 | 0.98 | 1.10 | 0.37 |  |
|  | 6 | 1.50 | 1.45 | 0.95 | 1.58 | 0.37 |  |
|  | 7 | 0.84 | 0.64 | 0.49 | 1.71 | 0.42 |  |
|  | 8 | 1.11 | 0.70 | 0.41 | 2.70 | 0.48 |  |
|  | 9 | 0.69 | 0.67 | 0.82 | 0.85 | 0.38 |  |
|  | 10 | 0.64 | 0.56 | 0.46 | 1.39 | 0.40 |  |
|  | 11 | 0.65 | 0.48 | 0.40 | 1.64 | 0.39 |  |
|  | 12 | 0.47 | 0.35 | 0.61 | 0.77 | 0.41 |  |
|  | 13 | 0.48 | 0.11 | 0.35 | 1.38 | 0.37 |  |
|  | 14 | 1.43 | 1.34 | 1.17 | 1.23 | 0.39 |  |
|  | 15 | 1.57 | 1.06 | 0.94 | 1.67 | 0.43 |  |
|  | 16 | 0.98 | 0.84 | 1.29 | 0.76 | 0.42 |  |
| 2 | 1 | 0.85 | 0.78 | 0.96 | 0.89 | 0.43 | 0.90±0.14 |
|  | 2 | 0.73 | 0.39 | 0.97 | 0.75 | 0.41 |  |
|  | 3 | 0.98 | 0.88 | 0.86 | 1.14 | 0.37 |  |
|  | 4 | 1.27 | 1.10 | 0.82 | 1.55 | 0.37 |  |
|  | 5 | 0.65 | 0.53 | 0.93 | 0.70 | 0.36 |  |
|  | 6 | 0.86 | 0.47 | 1.64 | 0.53 | 0.42 |  |
|  | 7 | 1.18 | 1.03 | 1.63 | 0.73 | 0.42 |  |
